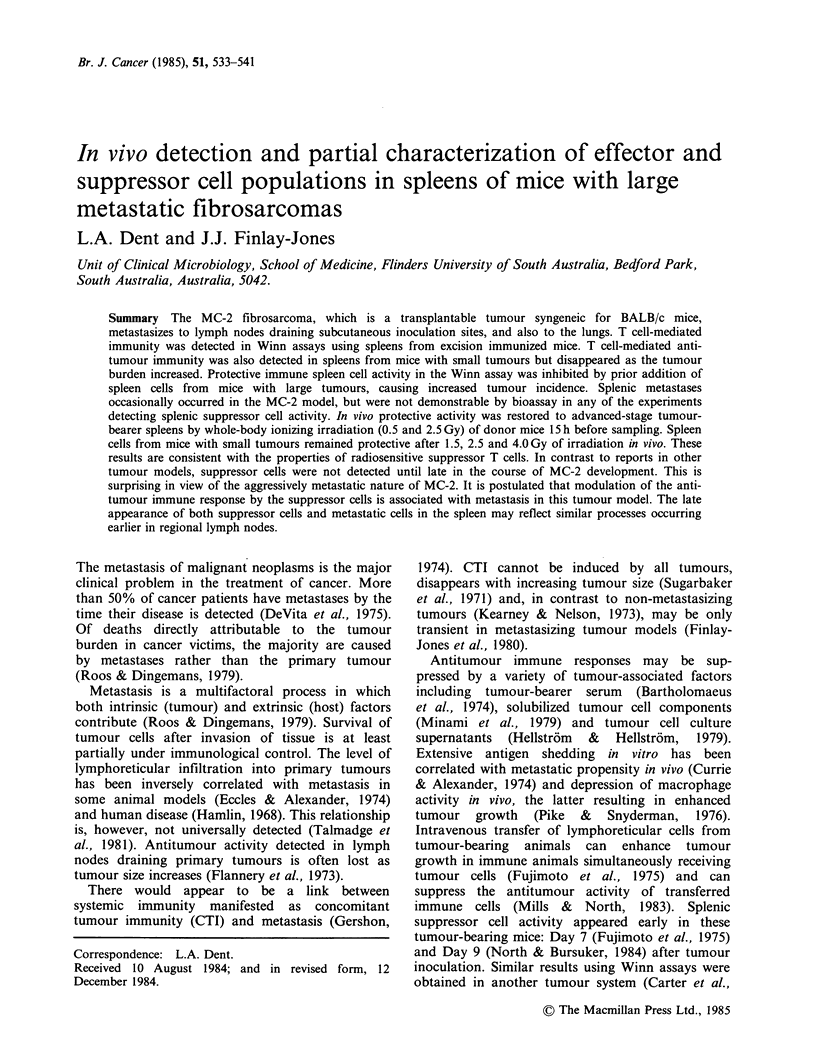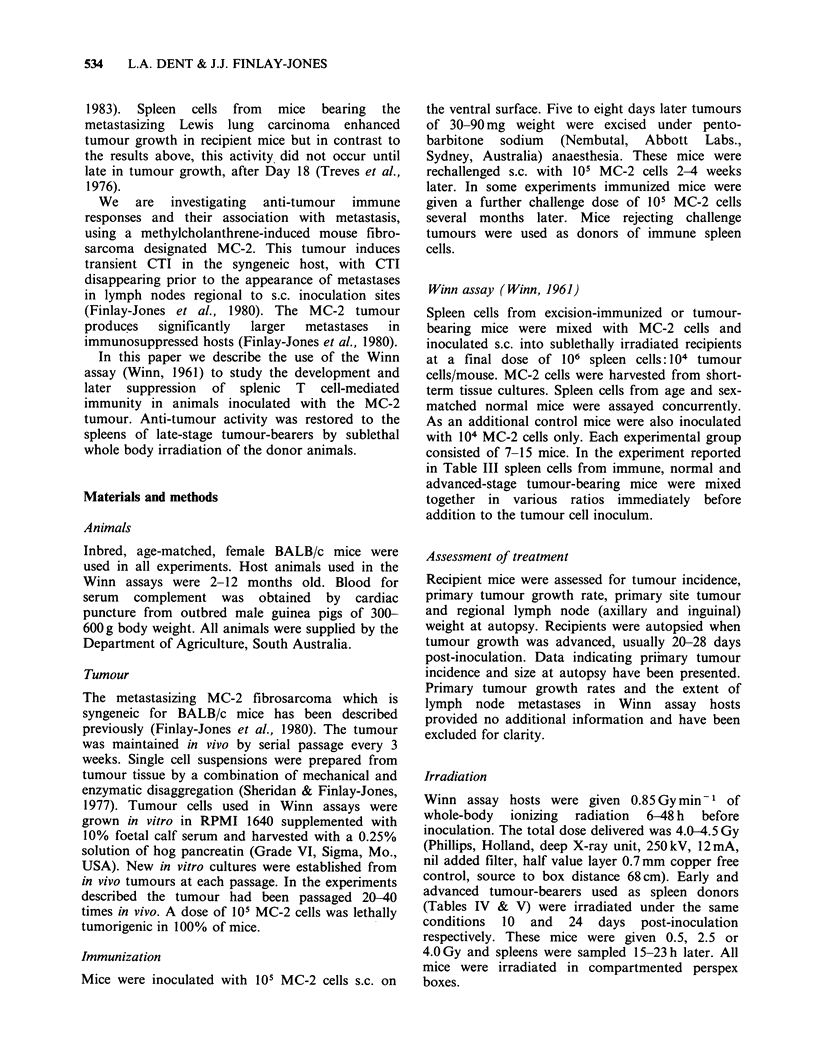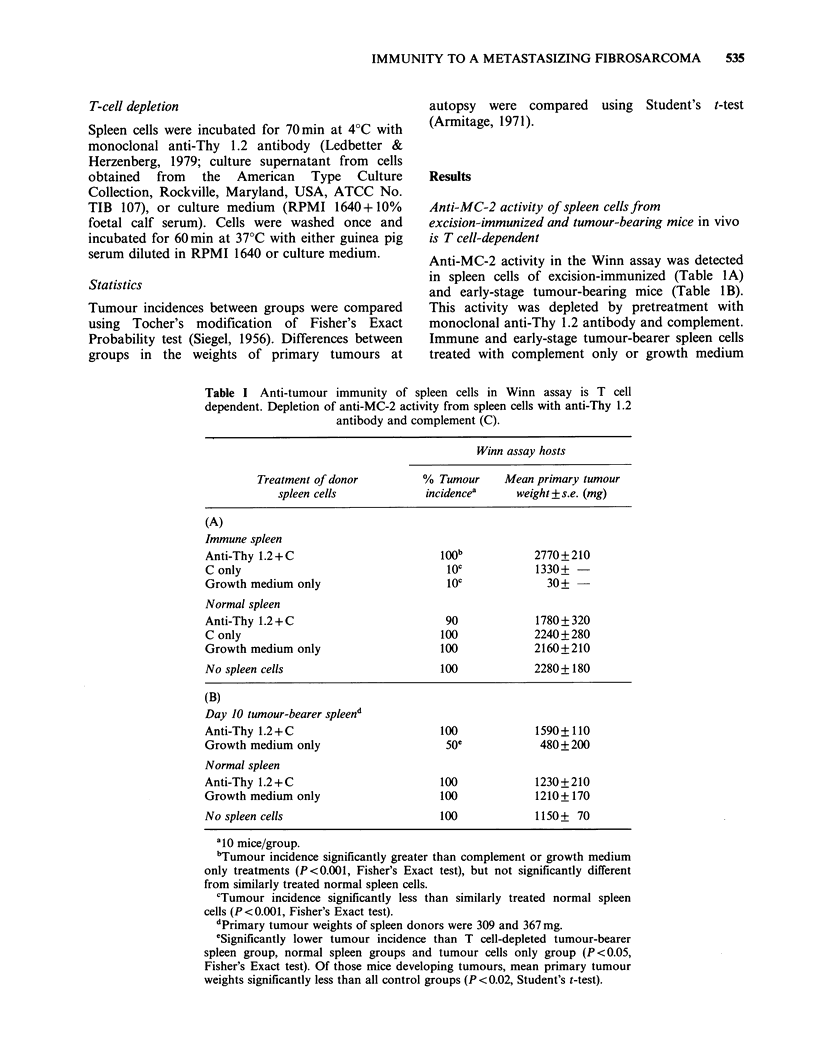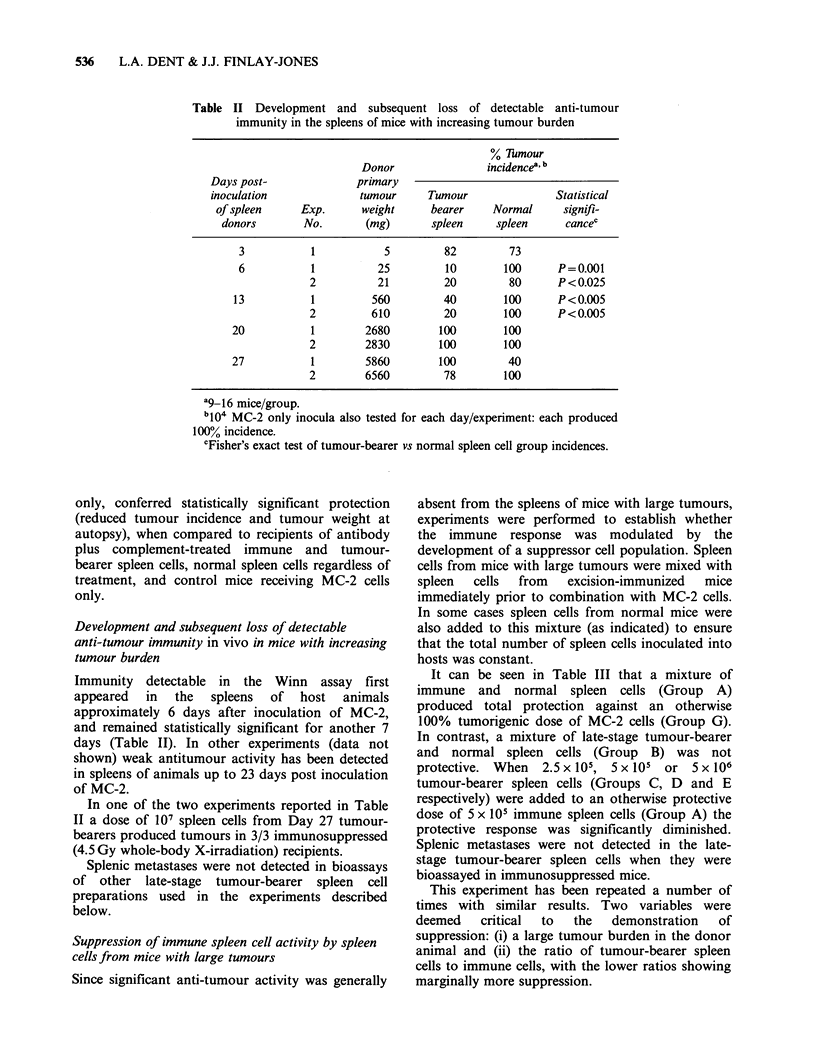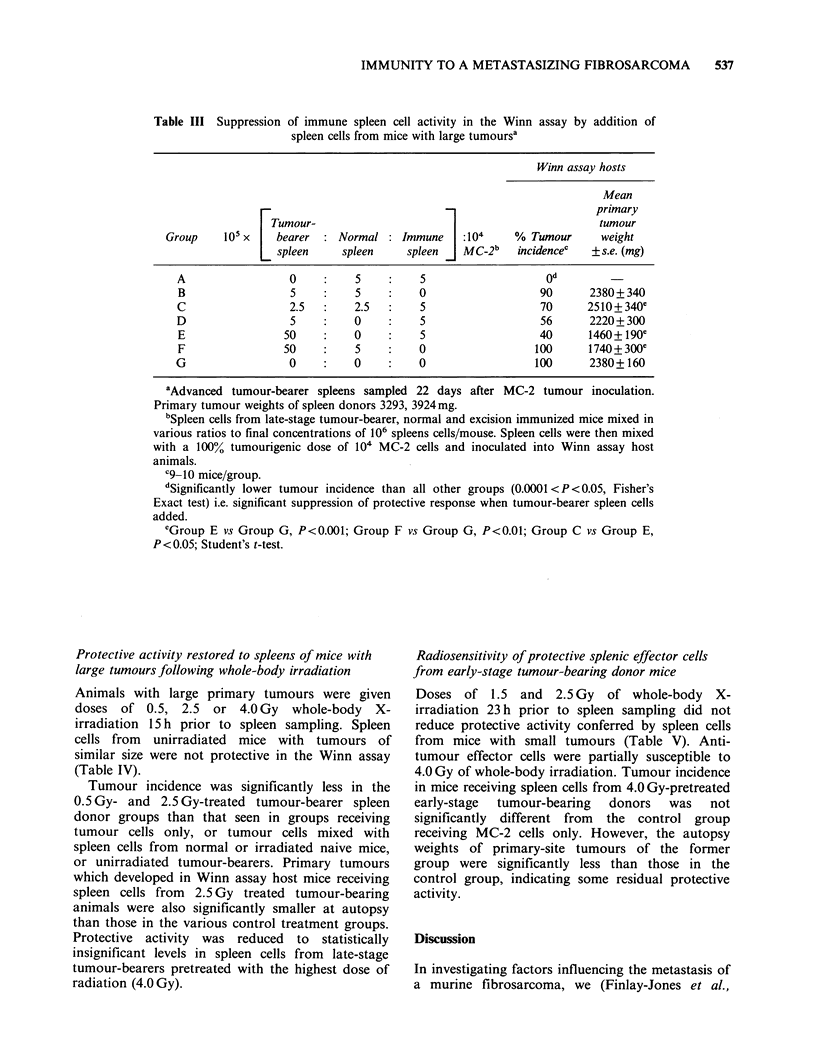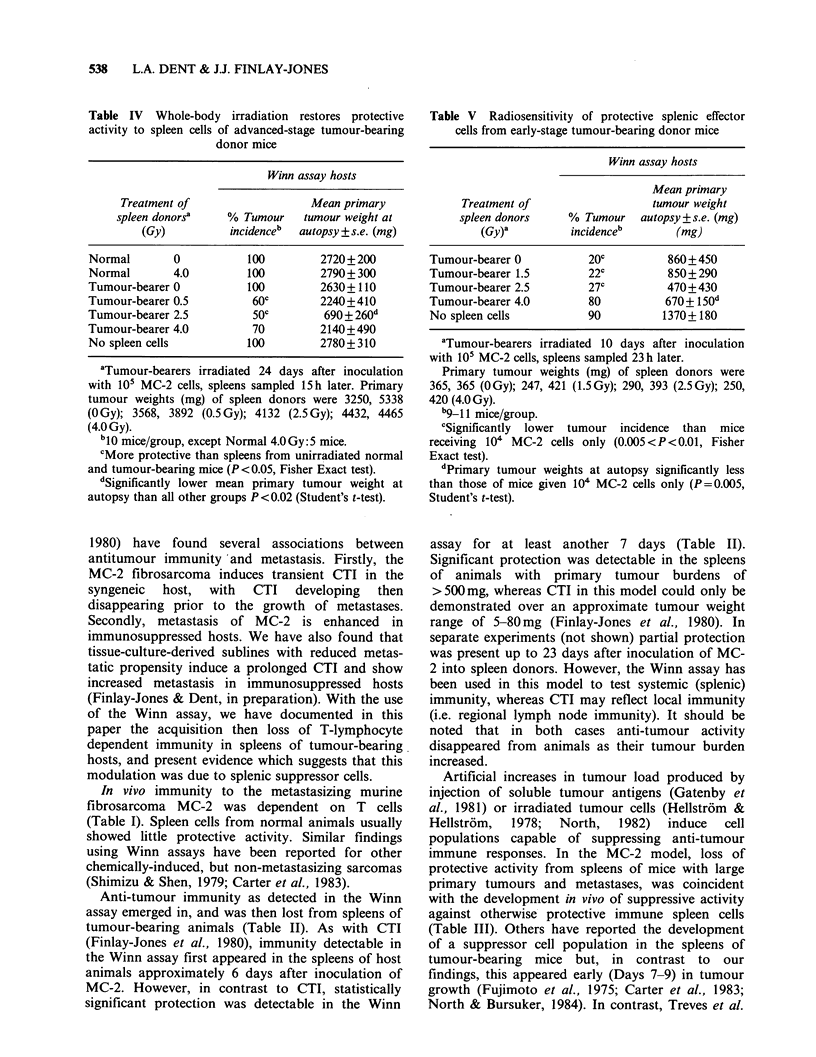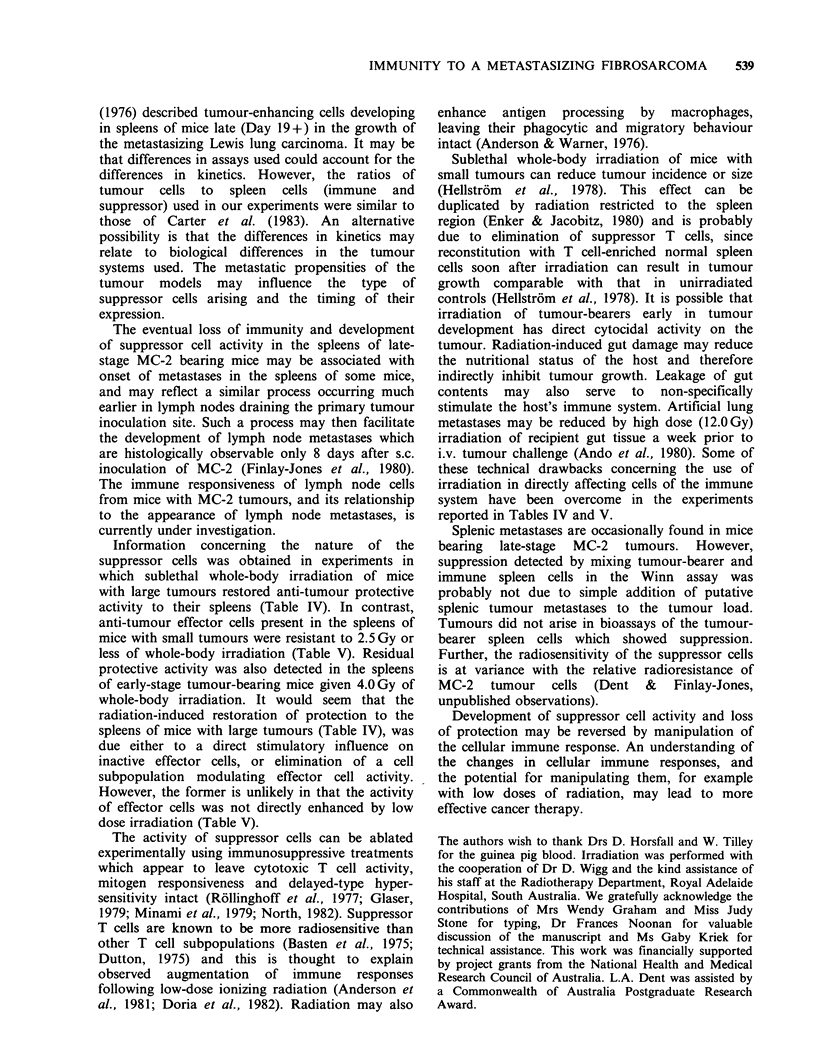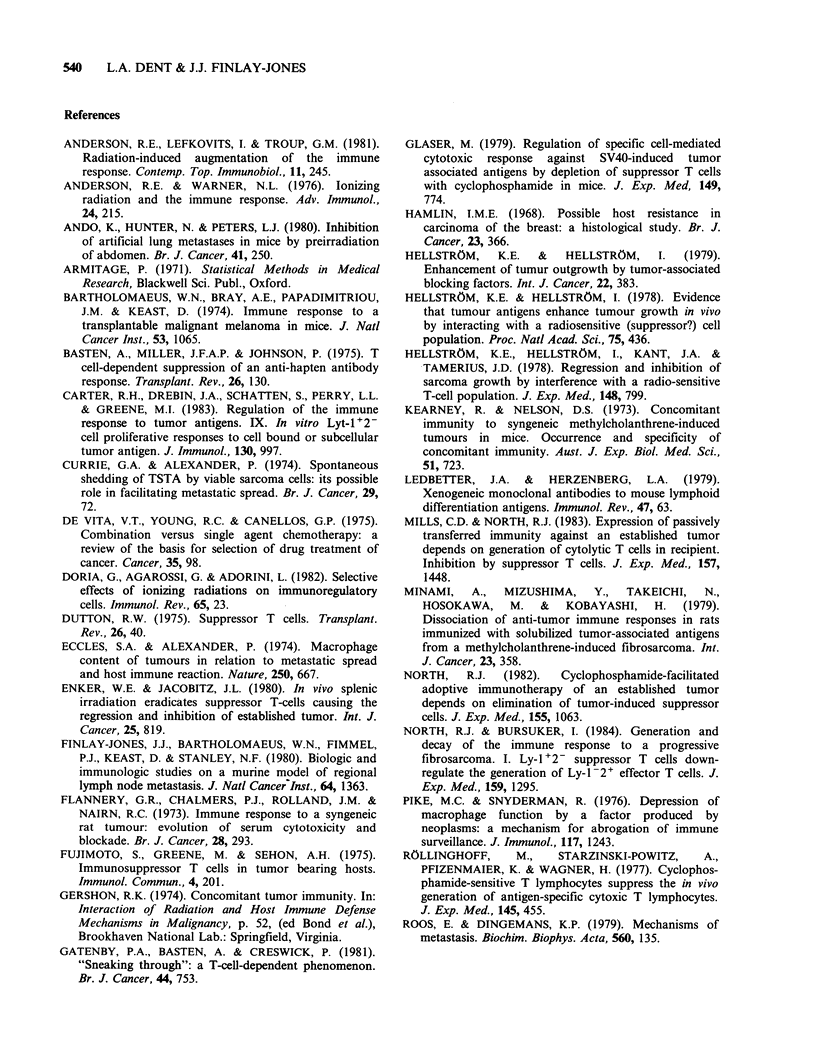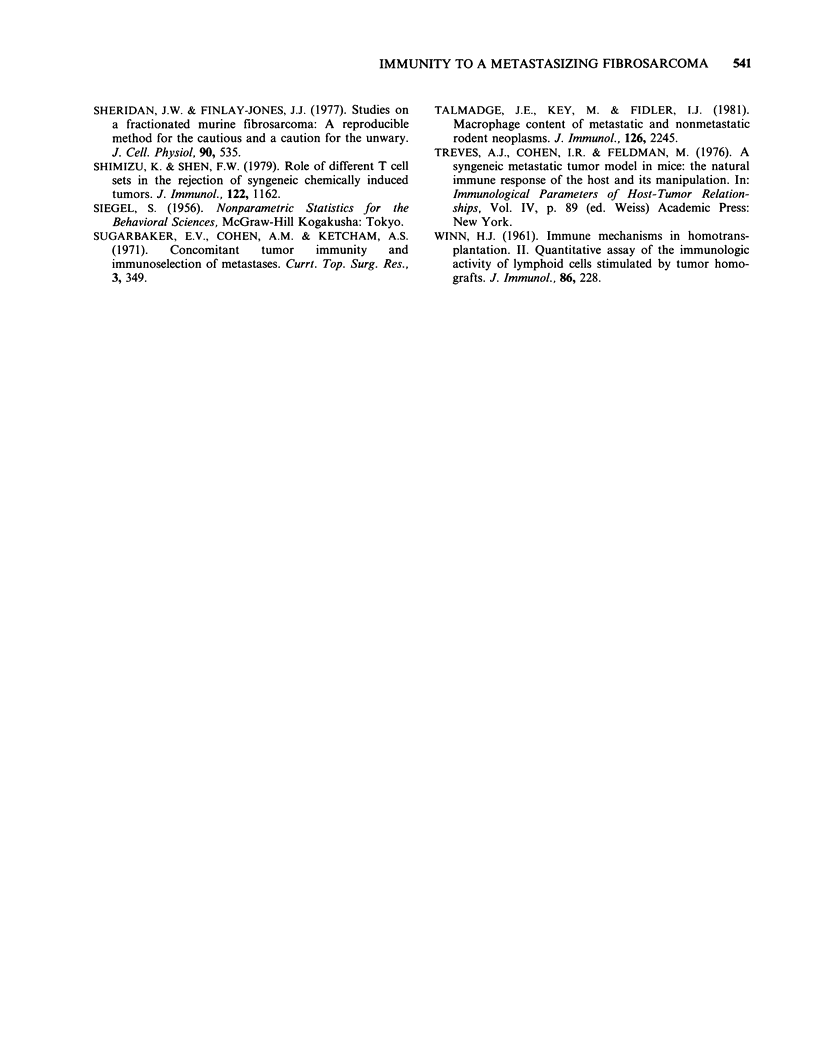# In vivo detection and partial characterization of effector and suppressor cell populations in spleens of mice with large metastatic fibrosarcomas.

**DOI:** 10.1038/bjc.1985.76

**Published:** 1985-04

**Authors:** L. A. Dent, J. J. Finlay-Jones

## Abstract

The MC-2 fibrosarcoma, which is a transplantable tumour syngeneic for BALB/c mice, metastasizes to lymph nodes draining subcutaneous inoculation sites, and also to the lungs. T cell-mediated immunity was detected in Winn assays using spleens from excision immunized mice. T cell-mediated anti-tumour immunity was also detected in spleens from mice with small tumours but disappeared as the tumour burden increased. Protective immune spleen cell activity in the Winn assay was inhibited by prior addition of spleen cells from mice with large tumours, causing increased tumour incidence. Splenic metastases occasionally occurred in the MC-2 model, but were not demonstrable by bioassay in any of the experiments detecting splenic suppressor cell activity. In vivo protective activity was restored to advanced-stage tumour-bearer spleens by whole-body ionizing irradiation (0.5 and 2.5 Gy) of donor mice 15 h before sampling. Spleen cells from mice with small tumours remained protective after 1.5, 2.5 and 4.0 Gy of irradiation in vivo. These results are consistent with the properties of radiosensitive suppressor T cells. In contrast to reports in other tumour models, suppressor cells were not detected until late in the course of MC-2 development. This is surprising in view of the aggressively metastatic nature of MC-2. It is postulated that modulation of the anti-tumour immune response by the suppressor cells is associated with metastasis in this tumour model. The late appearance of both suppressor cells and metastatic cells in the spleen may reflect similar processes occurring earlier in regional lymph nodes.


Br. J. Cancer (1985), 51, 533–541

### *In vivo* detection and partial characterization of effector and suppressor cell populations in spleens of mice with large metastatic fibrosarcomas

L.A. Dent and J.J. Finlay-Jones

*Unit of Clinical Microbiology, School of Medicine, Flinders University of South Australia, Bedford Park, South Australia, Australia, 5042.*

**Summary** The MC-2 fibrosarcoma, which is a transplantable tumour syngeneic for BALB/c mice, metastasizes to lymph nodes draining subcutaneous inoculation sites, and also to the lungs. T cell-mediated immunity was detected in Winn assays using spleens from excision immunized mice. T cell-mediated anti-tumour immunity was also detected in spleens from mice with small tumours but disappeared as the tumour burden increased. Protective immune spleen cell activity in the Winn assay was inhibited by prior addition of spleen cells from mice with large tumours, causing increased tumour incidence. Splenic metastases occasionally occurred in the MC-2 model, but were not demonstrable by bioassay in any of the experiments detecting splenic suppressor cell activity. *In vivo* protective activity was restored to advanced-stage tumour-bearer spleens by whole-body ionizing irradiation (0.5 and 2.5 Gy) of donor mice 15 h before sampling. Spleen cells from mice with small tumours remained protective after 1.5, 2.5 and 4.0 Gy of irradiation *in vivo*. These results are consistent with the properties of radiosensitive suppressor T cells. In contrast to reports in other tumour models, suppressor cells were not detected until late in the course of MC-2 development. This is surprising in view of the aggressively metastatic nature of MC-2. It is postulated that modulation of the anti-tumour immune response by the suppressor cells is associated with metastasis in this tumour model. The late appearance of both suppressor cells and metastatic cells in the spleen may reflect similar processes occurring earlier in regional lymph nodes.

The metastasis of malignant neoplasms is the major clinical problem in the treatment of cancer. More than 50% of cancer patients have metastases by the time their disease is detected (DeVita *et al.*, 1975). Of deaths directly attributable to the tumour burden in cancer victims, the majority are caused by metastases rather than the primary tumour (Roos & Dingemans, 1979).

Metastasis is a multifactoral process in which both intrinsic (tumour) and extrinsic (host) factors contribute (Roos & Dingemans, 1979). Survival of tumour cells after invasion of tissue is at least partially under immunological control. The level of lymphoreticular infiltration into primary tumours has been inversely correlated with metastasis in some animal models (Eccles & Alexander, 1974) and human disease (Hamlin, 1968). This relationship is, however, not universally detected (Talmadge *et al.*, 1981). Antitumour activity detected in lymph nodes draining primary tumours is often lost as tumour size increases (Flannery *et al.*, 1973).

There would appear to be a link between systemic immunity manifested as concomitant tumour immunity (CTI) and metastasis (Gershon, 1974). CTI cannot be induced by all tumours, disappears with increasing tumour size (Sugarbaker *et al.*, 1971) and, in contrast to non-metastasizing tumours (Kearney & Nelson, 1973), may be only transient in metastasizing tumour models (Finlay-Jones *et al.*, 1980).

Antitumour immune responses may be suppressed by a variety of tumour-associated factors including tumour-bearer serum (Bartholomaeus *et al.*, 1974), solubilized tumour cell components (Minami *et al.*, 1979) and tumour cell culture supernatants (Hellström & Hellström, 1979). Extensive antigen shedding *in vitro* has been correlated with metastatic propensity *in vivo* (Currie & Alexander, 1974) and depression of macrophage activity *in vivo*, the latter resulting in enhanced tumour growth (Pike & Snyderman, 1976). Intravenous transfer of lymphoreticular cells from tumour-bearing animals can enhance tumour growth in immune animals simultaneously receiving tumour cells (Fujimoto *et al.*, 1975) and can suppress the antitumour activity of transferred immune cells (Mills & North, 1983). Splenic suppressor cell activity appeared early in these tumour-bearing mice: Day 7 (Fujimoto *et al.*, 1975) and Day 9 (North & Bursuker, 1984) after tumour inoculation. Similar results using Winn assays were obtained in another tumour system (Carter *et al.*,

Correspondence: L.A. Dent.
Received 10 August 1984; and in revised form, 12 December 1984.

© The Macmillan Press Ltd., 1985

1983). Spleen cells from mice bearing the metastasizing Lewis lung carcinoma enhanced tumour growth in recipient mice but in contrast to the results above, this activity did not occur until late in tumour growth, after Day 18 (Treves *et al.*, 1976).

We are investigating anti-tumour immune responses and their association with metastasis, using a methylcholanthrene-induced mouse fibrosarcoma designated MC-2. This tumour induces transient CTI in the syngeneic host, with CTI disappearing prior to the appearance of metastases in lymph nodes regional to s.c. inoculation sites (Finlay-Jones *et al.*, 1980). The MC-2 tumour produces significantly larger metastases in immunosuppressed hosts (Finlay-Jones *et al.*, 1980).

In this paper we describe the use of the Winn assay (Winn, 1961) to study the development and later suppression of splenic T cell-mediated immunity in animals inoculated with the MC-2 tumour. Anti-tumour activity was restored to the spleens of late-stage tumour-bearers by sublethal whole body irradiation of the donor animals.

### Materials and methods

### *Animals*

Inbred, age-matched, female BALB/c mice were used in all experiments. Host animals used in the Winn assays were 2–12 months old. Blood for serum complement was obtained by cardiac puncture from outbred male guinea pigs of 300–600 g body weight. All animals were supplied by the Department of Agriculture, South Australia.

### *Tumour*

The metastasizing MC-2 fibrosarcoma which is syngeneic for BALB/c mice has been described previously (Finlay-Jones *et al.*, 1980). The tumour was maintained *in vivo* by serial passage every 3 weeks. Single cell suspensions were prepared from tumour tissue by a combination of mechanical and enzymatic disaggregation (Sheridan & Finlay-Jones, 1977). Tumour cells used in Winn assays were grown *in vitro* in RPMI 1640 supplemented with 10% foetal calf serum and harvested with a 0.25% solution of hog pancreatin (Grade VI, Sigma, Mo., USA). New *in vitro* cultures were established from *in vivo* tumours at each passage. In the experiments described the tumour had been passaged 20–40 times *in vivo*. A dose of $10^5$ MC-2 cells was lethally tumorigenic in 100% of mice.

### *Immunization*

Mice were inoculated with $10^5$ MC-2 cells s.c. on the ventral surface. Five to eight days later tumours of 30–90 mg weight were excised under pentobarbitone sodium (Nembutal, Abbott Labs., Sydney, Australia) anaesthesia. These mice were rechallenged s.c. with $10^5$ MC-2 cells 2–4 weeks later. In some experiments immunized mice were given a further challenge dose of $10^5$ MC-2 cells several months later. Mice rejecting challenge tumours were used as donors of immune spleen cells.

### *Winn assay (Winn, 1961)*

Spleen cells from excision-immunized or tumour-bearing mice were mixed with MC-2 cells and inoculated s.c. into sublethally irradiated recipients at a final dose of $10^6$ spleen cells:$10^4$ tumour cells/mouse. MC-2 cells were harvested from short-term tissue cultures. Spleen cells from age and sex-matched normal mice were assayed concurrently. As an additional control mice were also inoculated with $10^4$ MC-2 cells only. Each experimental group consisted of 7–15 mice. In the experiment reported in Table III spleen cells from immune, normal and advanced-stage tumour-bearing mice were mixed together in various ratios immediately before addition to the tumour cell inoculum.

### *Assessment of treatment*

Recipient mice were assessed for tumour incidence, primary tumour growth rate, primary site tumour and regional lymph node (axillary and inguinal) weight at autopsy. Recipients were autopsied when tumour growth was advanced, usually 20–28 days post-inoculation. Data indicating primary tumour incidence and size at autopsy have been presented. Primary tumour growth rates and the extent of lymph node metastases in Winn assay hosts provided no additional information and have been excluded for clarity.

### *Irradiation*

Winn assay hosts were given 0.85 Gy min$^{-1}$ of whole-body ionizing radiation 6–48 h before inoculation. The total dose delivered was 4.0–4.5 Gy (Phillips, Holland, deep X-ray unit, 250 kV, 12 mA, nil added filter, half value layer 0.7 mm copper free control, source to box distance 68 cm). Early and advanced tumour-bearers used as spleen donors (Tables IV & V) were irradiated under the same conditions 10 and 24 days post-inoculation respectively. These mice were given 0.5, 2.5 or 4.0 Gy and spleens were sampled 15–23 h later. All mice were irradiated in compartmented perspex boxes.

*T-cell depletion*

Spleen cells were incubated for 70 min at 4°C with monoclonal anti-Thy 1.2 antibody (Ledbetter & Herzenberg, 1979; culture supernatant from cells obtained from the American Type Culture Collection, Rockville, Maryland, USA, ATCC No. TIB 107), or culture medium (RPMI 1640+10% foetal calf serum). Cells were washed once and incubated for 60 min at 37°C with either guinea pig serum diluted in RPMI 1640 or culture medium.

*Statistics*

Tumour incidences between groups were compared using Tocher's modification of Fisher's Exact Probability test (Siegel, 1956). Differences between groups in the weights of primary tumours at autopsy were compared using Student's *t*-test (Armitage, 1971).

### Results

*Anti-MC-2 activity of spleen cells from excision-immunized and tumour-bearing mice* in vivo *is T cell-dependent*

Anti-MC-2 activity in the Winn assay was detected in spleen cells of excision-immunized (Table 1A) and early-stage tumour-bearing mice (Table 1B). This activity was depleted by pretreatment with monoclonal anti-Thy 1.2 antibody and complement. Immune and early-stage tumour-bearer spleen cells treated with complement only or growth medium

**Table I** Anti-tumour immunity of spleen cells in Winn assay is T cell dependent. Depletion of anti-MC-2 activity from spleen cells with anti-Thy 1.2 antibody and complement (C).

| | *Winn assay hosts* | |
|---|---|---|
| *Treatment of donor spleen cells* | *% Tumour incidence*[a] | *Mean primary tumour weight ± s.e. (mg)* |
| (A) | | |
| *Immune spleen* | | |
| Anti-Thy 1.2+C | 100[b] | 2770±210 |
| C only | 10[c] | 1330± — |
| Growth medium only | 10[c] | 30± — |
| *Normal spleen* | | |
| Anti-Thy 1.2+C | 90 | 1780±320 |
| C only | 100 | 2240±280 |
| Growth medium only | 100 | 2160±210 |
| *No spleen cells* | 100 | 2280±180 |
| (B) | | |
| *Day 10 tumour-bearer spleen*[d] | | |
| Anti-Thy 1.2+C | 100 | 1590±110 |
| Growth medium only | 50[e] | 480±200 |
| *Normal spleen* | | |
| Anti-Thy 1.2+C | 100 | 1230±210 |
| Growth medium only | 100 | 1210±170 |
| *No spleen cells* | 100 | 1150± 70 |

[a]10 mice/group.

[b]Tumour incidence significantly greater than complement or growth medium only treatments ($P<0.001$, Fisher's Exact test), but not significantly different from similarly treated normal spleen cells.

[c]Tumour incidence significantly less than similarly treated normal spleen cells ($P<0.001$, Fisher's Exact test).

[d]Primary tumour weights of spleen donors were 309 and 367 mg.

[e]Significantly lower tumour incidence than T cell-depleted tumour-bearer spleen group, normal spleen groups and tumour cells only group ($P<0.05$, Fisher's Exact test). Of those mice developing tumours, mean primary tumour weights significantly less than all control groups ($P<0.02$, Student's *t*-test).

**Table II** Development and subsequent loss of detectable anti-tumour immunity in the spleens of mice with increasing tumour burden

| *Days post-inoculation of spleen donors* | *Exp. No.* | *Donor primary tumour weight (mg)* | *% Tumour incidence*[a,b] *Tumour bearer spleen* | *Normal spleen* | *Statistical significance*[c] |
|---|---|---|---|---|---|
| 3 | 1 | 5 | 82 | 73 | |
| 6 | 1 | 25 | 10 | 100 | $P=0.001$ |
| | 2 | 21 | 20 | 80 | $P<0.025$ |
| 13 | 1 | 560 | 40 | 100 | $P<0.005$ |
| | 2 | 610 | 20 | 100 | $P<0.005$ |
| 20 | 1 | 2680 | 100 | 100 | |
| | 2 | 2830 | 100 | 100 | |
| 27 | 1 | 5860 | 100 | 40 | |
| | 2 | 6560 | 78 | 100 | |

[a]9–16 mice/group.
[b]$10^4$ MC-2 only inocula also tested for each day/experiment: each produced 100% incidence.
[c]Fisher's exact test of tumour-bearer *vs* normal spleen cell group incidences.

only, conferred statistically significant protection (reduced tumour incidence and tumour weight at autopsy), when compared to recipients of antibody plus complement-treated immune and tumour-bearer spleen cells, normal spleen cells regardless of treatment, and control mice receiving MC-2 cells only.

*Development and subsequent loss of detectable anti-tumour immunity* in vivo *in mice with increasing tumour burden*

Immunity detectable in the Winn assay first appeared in the spleens of host animals approximately 6 days after inoculation of MC-2, and remained statistically significant for another 7 days (Table II). In other experiments (data not shown) weak antitumour activity has been detected in spleens of animals up to 23 days post inoculation of MC-2.

In one of the two experiments reported in Table II a dose of $10^7$ spleen cells from Day 27 tumour-bearers produced tumours in 3/3 immunosuppressed (4.5 Gy whole-body X-irradiation) recipients.

Splenic metastases were not detected in bioassays of other late-stage tumour-bearer spleen cell preparations used in the experiments described below.

*Suppression of immune spleen cell activity by spleen cells from mice with large tumours*

Since significant anti-tumour activity was generally absent from the spleens of mice with large tumours, experiments were performed to establish whether the immune response was modulated by the development of a suppressor cell population. Spleen cells from mice with large tumours were mixed with spleen cells from excision-immunized mice immediately prior to combination with MC-2 cells. In some cases spleen cells from normal mice were also added to this mixture (as indicated) to ensure that the total number of spleen cells inoculated into hosts was constant.

It can be seen in Table III that a mixture of immune and normal spleen cells (Group A) produced total protection against an otherwise 100% tumorigenic dose of MC-2 cells (Group G). In contrast, a mixture of late-stage tumour-bearer and normal spleen cells (Group B) was not protective. When $2.5\times10^5$, $5\times10^5$ or $5\times10^6$ tumour-bearer spleen cells (Groups C, D and E respectively) were added to an otherwise protective dose of $5\times10^5$ immune spleen cells (Group A) the protective response was significantly diminished. Splenic metastases were not detected in the late-stage tumour-bearer spleen cells when they were bioassayed in immunosuppressed mice.

This experiment has been repeated a number of times with similar results. Two variables were deemed critical to the demonstration of suppression: (i) a large tumour burden in the donor animal and (ii) the ratio of tumour-bearer spleen cells to immune cells, with the lower ratios showing marginally more suppression.

**Table III** Suppression of immune spleen cell activity in the Winn assay by addition of spleen cells from mice with large tumours[a]

| *Group* | $10^5 \times$ [*Tumour-bearer spleen* | : | *Normal spleen* | : | *Immune spleen*] | $:10^4$ *MC-2*[b] | *Winn assay hosts* % *Tumour incidence*[c] | *Winn assay hosts* *Mean primary tumour weight* ±*s.e.* (*mg*) |
|---|---|---|---|---|---|---|---|---|
| A | 0 | : | 5 | : | 5 | | 0[d] | — |
| B | 5 | : | 5 | : | 0 | | 90 | 2380±340 |
| C | 2.5 | : | 2.5 | : | 5 | | 70 | 2510±340[e] |
| D | 5 | : | 0 | : | 5 | | 56 | 2220±300 |
| E | 50 | : | 0 | : | 5 | | 40 | 1460±190[e] |
| F | 50 | : | 5 | : | 0 | | 100 | 1740±300[e] |
| G | 0 | : | 0 | : | 0 | | 100 | 2380±160 |

[a]Advanced tumour-bearer spleens sampled 22 days after MC-2 tumour inoculation. Primary tumour weights of spleen donors 3293, 3924 mg.

[b]Spleen cells from late-stage tumour-bearer, normal and excision immunized mice mixed in various ratios to final concentrations of $10^6$ spleens cells/mouse. Spleen cells were then mixed with a 100% tumourigenic dose of $10^4$ MC-2 cells and inoculated into Winn assay host animals.

[c]9–10 mice/group.

[d]Significantly lower tumour incidence than all other groups ($0.0001 < P < 0.05$, Fisher's Exact test) i.e. significant suppression of protective response when tumour-bearer spleen cells added.

[e]Group E *vs* Group G, $P < 0.001$; Group F *vs* Group G, $P < 0.01$; Group C *vs* Group E, $P < 0.05$; Student's *t*-test.

*Protective activity restored to spleens of mice with large tumours following whole-body irradiation*

Animals with large primary tumours were given doses of 0.5, 2.5 or 4.0 Gy whole-body X-irradiation 15 h prior to spleen sampling. Spleen cells from unirradiated mice with tumours of similar size were not protective in the Winn assay (Table IV).

Tumour incidence was significantly less in the 0.5 Gy- and 2.5 Gy-treated tumour-bearer spleen donor groups than that seen in groups receiving tumour cells only, or tumour cells mixed with spleen cells from normal or irradiated naive mice, or unirradiated tumour-bearers. Primary tumours which developed in Winn assay host mice receiving spleen cells from 2.5 Gy treated tumour-bearing animals were also significantly smaller at autopsy than those in the various control treatment groups. Protective activity was reduced to statistically insignificant levels in spleen cells from late-stage tumour-bearers pretreated with the highest dose of radiation (4.0 Gy).

*Radiosensitivity of protective splenic effector cells from early-stage tumour-bearing donor mice*

Doses of 1.5 and 2.5 Gy of whole-body X-irradiation 23 h prior to spleen sampling did not reduce protective activity conferred by spleen cells from mice with small tumours (Table V). Anti-tumour effector cells were partially susceptible to 4.0 Gy of whole-body irradiation. Tumour incidence in mice receiving spleen cells from 4.0 Gy-pretreated early-stage tumour-bearing donors was not significantly different from the control group receiving MC-2 cells only. However, the autopsy weights of primary-site tumours of the former group were significantly less than those in the control group, indicating some residual protective activity.

### Discussion

In investigating factors influencing the metastasis of a murine fibrosarcoma, we (Finlay-Jones *et al.*,

**Table IV** Whole-body irradiation restores protective activity to spleen cells of advanced-stage tumour-bearing donor mice

| *Treatment of spleen donors*[a] *(Gy)* | *Winn assay hosts* *% Tumour incidence*[b] | *Mean primary tumour weight at autopsy* ± *s.e. (mg)* |
|---|---|---|
| Normal 0 | 100 | 2720±200 |
| Normal 4.0 | 100 | 2790±300 |
| Tumour-bearer 0 | 100 | 2630±110 |
| Tumour-bearer 0.5 | 60[c] | 2240±410 |
| Tumour-bearer 2.5 | 50[c] | 690±260[d] |
| Tumour-bearer 4.0 | 70 | 2140±490 |
| No spleen cells | 100 | 2780±310 |

[a]Tumour-bearers irradiated 24 days after inoculation with $10^5$ MC-2 cells, spleens sampled 15 h later. Primary tumour weights (mg) of spleen donors were 3250, 5338 (0 Gy); 3568, 3892 (0.5 Gy); 4132 (2.5 Gy); 4432, 4465 (4.0 Gy).

[b]10 mice/group, except Normal 4.0 Gy: 5 mice.

[c]More protective than spleens from unirradiated normal and tumour-bearing mice ($P<0.05$, Fisher Exact test).

[d]Significantly lower mean primary tumour weight at autopsy than all other groups $P<0.02$ (Student's *t*-test).

**Table V** Radiosensitivity of protective splenic effector cells from early-stage tumour-bearing donor mice

| *Treatment of spleen donors (Gy)*[a] | *Winn assay hosts* *% Tumour incidence*[b] | *Mean primary tumour weight autopsy* ± *s.e. (mg) (mg)* |
|---|---|---|
| Tumour-bearer 0 | 20[c] | 860±450 |
| Tumour-bearer 1.5 | 22[c] | 850±290 |
| Tumour-bearer 2.5 | 27[c] | 470±430 |
| Tumour-bearer 4.0 | 80 | 670±150[d] |
| No spleen cells | 90 | 1370±180 |

[a]Tumour-bearers irradiated 10 days after inoculation with $10^5$ MC-2 cells, spleens sampled 23 h later.

Primary tumour weights (mg) of spleen donors were 365, 365 (0 Gy); 247, 421 (1.5 Gy); 290, 393 (2.5 Gy); 250, 420 (4.0 Gy).

[b]9–11 mice/group.

[c]Significantly lower tumour incidence than mice receiving $10^4$ MC-2 cells only ($0.005<P<0.01$, Fisher Exact test).

[d]Primary tumour weights at autopsy significantly less than those of mice given $10^4$ MC-2 cells only ($P=0.005$, Student's *t*-test).

1980) have found several associations between antitumour immunity and metastasis. Firstly, the MC-2 fibrosarcoma induces transient CTI in the syngeneic host, with CTI developing then disappearing prior to the growth of metastases. Secondly, metastasis of MC-2 is enhanced in immunosuppressed hosts. We have also found that tissue-culture-derived sublines with reduced metastatic propensity induce a prolonged CTI and show increased metastasis in immunosuppressed hosts (Finlay-Jones & Dent, in preparation). With the use of the Winn assay, we have documented in this paper the acquisition then loss of T-lymphocyte dependent immunity in spleens of tumour-bearing hosts, and present evidence which suggests that this modulation was due to splenic suppressor cells.

*In vivo* immunity to the metastasizing murine fibrosarcoma MC-2 was dependent on T cells (Table I). Spleen cells from normal animals usually showed little protective activity. Similar findings using Winn assays have been reported for other chemically-induced, but non-metastasizing sarcomas (Shimizu & Shen, 1979; Carter *et al.*, 1983).

Anti-tumour immunity as detected in the Winn assay emerged in, and was then lost from spleens of tumour-bearing animals (Table II). As with CTI (Finlay-Jones *et al.*, 1980), immunity detectable in the Winn assay first appeared in the spleens of host animals approximately 6 days after inoculation of MC-2. However, in contrast to CTI, statistically significant protection was detectable in the Winn assay for at least another 7 days (Table II). Significant protection was detectable in the spleens of animals with primary tumour burdens of >500 mg, whereas CTI in this model could only be demonstrated over an approximate tumour weight range of 5–80 mg (Finlay-Jones *et al.*, 1980). In separate experiments (not shown) partial protection was present up to 23 days after inoculation of MC-2 into spleen donors. However, the Winn assay has been used in this model to test systemic (splenic) immunity, whereas CTI may reflect local immunity (i.e. regional lymph node immunity). It should be noted that in both cases anti-tumour activity disappeared from animals as their tumour burden increased.

Artificial increases in tumour load produced by injection of soluble tumour antigens (Gatenby *et al.*, 1981) or irradiated tumour cells (Hellström & Hellström, 1978; North, 1982) induce cell populations capable of suppressing anti-tumour immune responses. In the MC-2 model, loss of protective activity from spleens of mice with large primary tumours and metastases, was coincident with the development *in vivo* of suppressive activity against otherwise protective immune spleen cells (Table III). Others have reported the development of a suppressor cell population in the spleens of tumour-bearing mice but, in contrast to our findings, this appeared early (Days 7–9) in tumour growth (Fujimoto *et al.*, 1975; Carter *et al.*, 1983; North & Bursuker, 1984). In contrast, Treves *et al.*

(1976) described tumour-enhancing cells developing in spleens of mice late (Day 19+) in the growth of the metastasizing Lewis lung carcinoma. It may be that differences in assays used could account for the differences in kinetics. However, the ratios of tumour cells to spleen cells (immune and suppressor) used in our experiments were similar to those of Carter *et al.* (1983). An alternative possibility is that the differences in kinetics may relate to biological differences in the tumour systems used. The metastatic propensities of the tumour models may influence the type of suppressor cells arising and the timing of their expression.

The eventual loss of immunity and development of suppressor cell activity in the spleens of late-stage MC-2 bearing mice may be associated with onset of metastases in the spleens of some mice, and may reflect a similar process occurring much earlier in lymph nodes draining the primary tumour inoculation site. Such a process may then facilitate the development of lymph node metastases which are histologically observable only 8 days after s.c. inoculation of MC-2 (Finlay-Jones *et al.*, 1980). The immune responsiveness of lymph node cells from mice with MC-2 tumours, and its relationship to the appearance of lymph node metastases, is currently under investigation.

Information concerning the nature of the suppressor cells was obtained in experiments in which sublethal whole-body irradiation of mice with large tumours restored anti-tumour protective activity to their spleens (Table IV). In contrast, anti-tumour effector cells present in the spleens of mice with small tumours were resistant to 2.5 Gy or less of whole-body irradiation (Table V). Residual protective activity was also detected in the spleens of early-stage tumour-bearing mice given 4.0 Gy of whole-body irradiation. It would seem that the radiation-induced restoration of protection to the spleens of mice with large tumours (Table IV), was due either to a direct stimulatory influence on inactive effector cells, or elimination of a cell subpopulation modulating effector cell activity. However, the former is unlikely in that the activity of effector cells was not directly enhanced by low dose irradiation (Table V).

The activity of suppressor cells can be ablated experimentally using immunosuppressive treatments which appear to leave cytotoxic T cell activity, mitogen responsiveness and delayed-type hypersensitivity intact (Röllinghoff *et al.*, 1977; Glaser, 1979; Minami *et al.*, 1979; North, 1982). Suppressor T cells are known to be more radiosensitive than other T cell subpopulations (Basten *et al.*, 1975; Dutton, 1975) and this is thought to explain observed augmentation of immune responses following low-dose ionizing radiation (Anderson *et al.*, 1981; Doria *et al.*, 1982). Radiation may also enhance antigen processing by macrophages, leaving their phagocytic and migratory behaviour intact (Anderson & Warner, 1976).

Sublethal whole-body irradiation of mice with small tumours can reduce tumour incidence or size (Hellström *et al.*, 1978). This effect can be duplicated by radiation restricted to the spleen region (Enker & Jacobitz, 1980) and is probably due to elimination of suppressor T cells, since reconstitution with T cell-enriched normal spleen cells soon after irradiation can result in tumour growth comparable with that in unirradiated controls (Hellström *et al.*, 1978). It is possible that irradiation of tumour-bearers early in tumour development has direct cytocidal activity on the tumour. Radiation-induced gut damage may reduce the nutritional status of the host and therefore indirectly inhibit tumour growth. Leakage of gut contents may also serve to non-specifically stimulate the host's immune system. Artificial lung metastases may be reduced by high dose (12.0 Gy) irradiation of recipient gut tissue a week prior to i.v. tumour challenge (Ando *et al.*, 1980). Some of these technical drawbacks concerning the use of irradiation in directly affecting cells of the immune system have been overcome in the experiments reported in Tables IV and V.

Splenic metastases are occasionally found in mice bearing late-stage MC-2 tumours. However, suppression detected by mixing tumour-bearer and immune spleen cells in the Winn assay was probably not due to simple addition of putative splenic tumour metastases to the tumour load. Tumours did not arise in bioassays of the tumour-bearer spleen cells which showed suppression. Further, the radiosensitivity of the suppressor cells is at variance with the relative radioresistance of MC-2 tumour cells (Dent & Finlay-Jones, unpublished observations).

Development of suppressor cell activity and loss of protection may be reversed by manipulation of the cellular immune response. An understanding of the changes in cellular immune responses, and the potential for manipulating them, for example with low doses of radiation, may lead to more effective cancer therapy.

The authors wish to thank Drs D. Horsfall and W. Tilley for the guinea pig blood. Irradiation was performed with the cooperation of Dr D. Wigg and the kind assistance of his staff at the Radiotherapy Department, Royal Adelaide Hospital, South Australia. We gratefully acknowledge the contributions of Mrs Wendy Graham and Miss Judy Stone for typing, Dr Frances Noonan for valuable discussion of the manuscript and Ms Gaby Kriek for technical assistance. This work was financially supported by project grants from the National Health and Medical Research Council of Australia. L.A. Dent was assisted by a Commonwealth of Australia Postgraduate Research Award.

### References

ANDERSON, R.E., LEFKOVITS, I. & TROUP, G.M. (1981). Radiation-induced augmentation of the immune response. *Contemp. Top. Immunobiol.*, **11**, 245.

ANDERSON, R.E. & WARNER, N.L. (1976). Ionizing radiation and the immune response. *Adv. Immunol.*, **24**, 215.

ANDO, K., HUNTER, N. & PETERS, L.J. (1980). Inhibition of artificial lung metastases in mice by preirradiation of abdomen. *Br. J. Cancer*, **41**, 250.

ARMITAGE, P. (1971). *Statistical Methods in Medical Research*, Blackwell Sci. Publ., Oxford.

BARTHOLOMAEUS, W.N., BRAY, A.E., PAPADIMITRIOU, J.M. & KEAST, D. (1974). Immune response to a transplantable malignant melanoma in mice. *J. Natl Cancer Inst.*, **53**, 1065.

BASTEN, A., MILLER, J.F.A.P. & JOHNSON, P. (1975). T cell-dependent suppression of an anti-hapten antibody response. *Transplant. Rev.*, **26**, 130.

CARTER, R.H., DREBIN, J.A., SCHATTEN, S., PERRY, L.L. & GREENE, M.I. (1983). Regulation of the immune response to tumor antigens. IX. *In vitro* Lyt-$1^+2^-$ cell proliferative responses to cell bound or subcellular tumor antigen. *J. Immunol.*, **130**, 997.

CURRIE, G.A. & ALEXANDER, P. (1974). Spontaneous shedding of TSTA by viable sarcoma cells: its possible role in facilitating metastatic spread. *Br. J. Cancer*, **29**, 72.

DE VITA, V.T., YOUNG, R.C. & CANELLOS, G.P. (1975). Combination versus single agent chemotherapy: a review of the basis for selection of drug treatment of cancer. *Cancer*, **35**, 98.

DORIA, G., AGAROSSI, G. & ADORINI, L. (1982). Selective effects of ionizing radiations on immunoregulatory cells. *Immunol. Rev.*, **65**, 23.

DUTTON, R.W. (1975). Suppressor T cells. *Transplant. Rev.*, **26**, 40.

ECCLES, S.A. & ALEXANDER, P. (1974). Macrophage content of tumours in relation to metastatic spread and host immune reaction. *Nature*, **250**, 667.

ENKER, W.E. & JACOBITZ, J.L. (1980). *In vivo* splenic irradiation eradicates suppressor T-cells causing the regression and inhibition of established tumor. *Int. J. Cancer*, **25**, 819.

FINLAY-JONES, J.J., BARTHOLOMAEUS, W.N., FIMMEL, P.J., KEAST, D. & STANLEY, N.F. (1980). Biologic and immunologic studies on a murine model of regional lymph node metastasis. *J. Natl Cancer Inst.*, **64**, 1363.

FLANNERY, G.R., CHALMERS, P.J., ROLLAND, J.M. & NAIRN, R.C. (1973). Immune response to a syngeneic rat tumour: evolution of serum cytotoxicity and blockade. *Br. J. Cancer*, **28**, 293.

FUJIMOTO, S., GREENE, M. & SEHON, A.H. (1975). Immunosuppressor T cells in tumor bearing hosts. *Immunol. Commun.*, **4**, 201.

GERSHON, R.K. (1974). Concomitant tumor immunity. In: *Interaction of Radiation and Host Immune Defense Mechanisms in Malignancy*, p. 52, (ed Bond *et al.*), Brookhaven National Lab.: Springfield, Virginia.

GATENBY, P.A., BASTEN, A. & CRESWICK, P. (1981). "Sneaking through": a T-cell-dependent phenomenon. *Br. J. Cancer*, **44**, 753.

GLASER, M. (1979). Regulation of specific cell-mediated cytotoxic response against SV40-induced tumor associated antigens by depletion of suppressor T cells with cyclophosphamide in mice. *J. Exp. Med*, **149**, 774.

HAMLIN, I.M.E. (1968). Possible host resistance in carcinoma of the breast: a histological study. *Br. J. Cancer*, **23**, 366.

HELLSTRÖM, K.E. & HELLSTRÖM, I. (1979). Enhancement of tumur outgrowth by tumor-associated blocking factors. *Int. J. Cancer*, **22**, 383.

HELLSTRÖM, K.E. & HELLSTRÖM, I. (1978). Evidence that tumour antigens enhance tumour growth *in vivo* by interacting with a radiosensitive (suppressor?) cell population. *Proc. Natl Acad. Sci.*, **75**, 436.

HELLSTRÖM, K.E., HELLSTRÖM, I., KANT, J.A. & TAMERIUS, J.D. (1978). Regression and inhibition of sarcoma growth by interference with a radio-sensitive T-cell population. *J. Exp. Med.*, **148**, 799.

KEARNEY, R. & NELSON, D.S. (1973). Concomitant immunity to syngeneic methylcholanthrene-induced tumours in mice. Occurrence and specificity of concomitant immunity. *Aust. J. Exp. Biol. Med. Sci.*, **51**, 723.

LEDBETTER, J.A. & HERZENBERG, L.A. (1979). Xenogeneic monoclonal antibodies to mouse lymphoid differentiation antigens. *Immunol. Rev.*, **47**, 63.

MILLS, C.D. & NORTH, R.J. (1983). Expression of passively transferred immunity against an established tumor depends on generation of cytolytic T cells in recipient. Inhibition by suppressor T cells. *J. Exp. Med.*, **157**, 1448.

MINAMI, A., MIZUSHIMA, Y., TAKEICHI, N., HOSOKAWA, M. & KOBAYASHI, H. (1979). Dissociation of anti-tumor immune responses in rats immunized with solubilized tumor-associated antigens from a methylcholanthrene-induced fibrosarcoma. *Int. J. Cancer*, **23**, 358.

NORTH, R.J. (1982). Cyclophosphamide-facilitated adoptive immunotherapy of an established tumor depends on elimination of tumor-induced suppressor cells. *J. Exp. Med.*, **155**, 1063.

NORTH, R.J. & BURSUKER, I. (1984). Generation and decay of the immune response to a progressive fibrosarcoma. I. Ly-$1^+2^-$ suppressor T cells down-regulate the generation of Ly-$1^-2^+$ effector T cells. *J. Exp. Med.*, **159**, 1295.

PIKE, M.C. & SNYDERMAN, R. (1976). Depression of macrophage function by a factor produced by neoplasms: a mechanism for abrogation of immune surveillance. *J. Immunol.*, **117**, 1243.

RÖLLINGHOFF, M., STARZINSKI-POWITZ, A., PFIZENMAIER, K. & WAGNER, H. (1977). Cyclophosphamide-sensitive T lymphocytes suppress the *in vivo* generation of antigen-specific cytoxic T lymphocytes. *J. Exp. Med.*, **145**, 455.

ROOS, E. & DINGEMANS, K.P. (1979). Mechanisms of metastasis. *Biochim. Biophys. Acta*, **560**, 135.

SHERIDAN, J.W. & FINLAY-JONES, J.J. (1977). Studies on a fractionated murine fibrosarcoma: A reproducible method for the cautious and a caution for the unwary. *J. Cell. Physiol*, **90,** 535.

SHIMIZU, K. & SHEN, F.W. (1979). Role of different T cell sets in the rejection of syngeneic chemically induced tumors. *J. Immunol.*, **122,** 1162.

SIEGEL, S. (1956). *Nonparametric Statistics for the Behavioral Sciences,* McGraw-Hill Kogakusha: Tokyo.

SUGARBAKER, E.V., COHEN, A.M. & KETCHAM, A.S. (1971). Concomitant tumor immunity and immunoselection of metastases. *Currt. Top. Surg. Res.*, **3,** 349.

TALMADGE, J.E., KEY, M. & FIDLER, I.J. (1981). Macrophage content of metastatic and nonmetastatic rodent neoplasms. *J. Immunol.*, **126,** 2245.

TREVES, A.J., COHEN, I.R. & FELDMAN, M. (1976). A syngeneic metastatic tumor model in mice: the natural immune response of the host and its manipulation. In: *Immunological Parameters of Host-Tumor Relationships,* Vol. IV, p. 89 (ed. Weiss) Academic Press: New York.

WINN, H.J. (1961). Immune mechanisms in homotransplantation. II. Quantitative assay of the immunologic activity of lymphoid cells stimulated by tumor homografts. *J. Immunol.*, **86,** 228.